# Managerial perceptions of core competencies for healthcare middle managers in Bahrain and Saudi Arabia: a qualitative study

**DOI:** 10.5116/ijme.6957.fb46

**Published:** 2026-01-21

**Authors:** Ahmed Mohamed Al Ansari

**Affiliations:** 1 Royal College of Surgeons in Ireland - Medical University of Bahrain (RCSI-MUB)

**Keywords:** Healthcare middle managers, managerial competencies, leadership development, qualitative study

## Abstract

**Objectives:**

This study aimed to identify and compare the perceptions of senior, middle, and frontline managers regarding the core competencies required for effective healthcare middle managers in Bahrain and Saudi Arabia. It also explored how these competencies can inform competency-based leadership development programs globally.

**Methods:**

A qualitative descriptive design was adopted. Twenty-seven participants from healthcare and medical education institutions in Bahrain and Saudi Arabia were purposively selected across three hierarchical levels: senior (n = 6, 22%), middle (n = 10, 37%), and frontline (n = 11, 41%). Semi-structured interviews were conducted virtually, audio-recorded, transcribed verbatim, and analyzed thematically using Braun and Clarke’s six-step framework. Credibility was strengthened through member checking, peer debriefing, and an audit trail.

**Results:**

Five overarching competency domains emerged in the study. They include personality, managerial skills, work ethics, mental ability and interaction. Personality, integrity, and organizational ability were the most frequently cited traits (93%). Managerial and social competencies such as time management (90%), teamwork (83%), communication (70%), and decision-making (60%) were emphasized across all managerial levels. Senior managers prioritized strategic agility and ethics, whereas frontline managers stressed interaction and communication. These patterns reflect the dual operational and relational demands of middle management in healthcare.

**Conclusions:**

Successful healthcare middle managers require a balanced integration of technical proficiency, ethical integrity, interpersonal competence, and adaptability. Embedding these domains into competency-based leadership training could enhance organizational performance and strengthen healthcare governance across the region.

## Introduction

The need for world-class, responsive, leadership and management for healthcare systems cannot be over-emphasized. The healthcare system is challenged by persistent increase in the demand for health care services, driven by rapid population growth and a high incidence of non-communicable diseases (NCDs).^1^ Additional demographic pressure such as a rising elderly population, as well as socioeconomic reforms, liberal urbanization, and lifestyle changes, significantly impact the overall health status of the population.^2^^,^^3^ These conditions have also resulted in the need for innovative and adaptive leadership and management across health institutions to navigate these complex challenges effectively.^3^ The Kingdom of Saudi Arabia (KSA) and the Kingdom of Bahrain (KB) are monarchies and are currently undergoing a significant transformation from a government-controlled healthcare system to a more self-sustained, autonomous model.^4^ The magnitude and complexity of this transformation require effective leadership at all levels of management (senior, middle and frontline).

Effective healthcare leadership depends on the ability of managers at all levels to translate strategy into coordinated action.^5^^-^^8^ Among these tiers, middle managers (MMs) serve as the vital bridge between strategic leadership and frontline service delivery. MMs are individuals positioned between senior executives and frontline staff who are responsible for interpreting strategy, coordinating operations, and guiding teams. Senior and frontline managers refer respectively to strategic decision-makers (e.g., deans, CEOs) and those reporting to MMs (e.g., clinical or administrative supervisors). MMs operationalize institutional goals, ensure communication across hierarchical levels, and maintain team performance under complex and evolving healthcare conditions. ^9^^-^^12^The competency of healthcare middle managers has been recognized as a decisive factor influencing organizational quality, staff satisfaction, and patient outcomes.^9^^,^^11^^,^^12^

In the management and leadership literature, skills are task-specific abilities that can be learned or developed (e.g., communication or problem-solving).^13^ Traits and personal characteristics refer to enduring personal dispositions such as integrity, confidence, or adaptability that influence behavior across situations.^14^ Competencies, in contrast, represent the integrated combination of knowledge, skills, attitudes, and traits that together enable effective performance in a specific role or context.^15^^,^^16^ Thus, in this study, the term competency is used as an overarching construct that encompasses both technical skills and personal traits required for successful middle management in healthcare.

In the Kingdom of Saudi Arabia (KSA) and the Kingdom of Bahrain (KB), national visions and policy reforms have emphasized health-system autonomy, efficiency, and quality improvement. These reforms demand managers who can navigate new governance structures and lead teams through change.^17^^,^^18^ Yet, despite the critical role of MMs in implementing these transformations, there remains a gap in understanding what competencies define successful middle management in these settings. Existing research in these regions has focused largely on senior leadership development or physician management training,^17^^,^^18^ with little exploration of the competency expectations across hierarchical levels or comparative perspectives between countries.

This study aimed to identify and compare the perceptions of senior, middle, and frontline managers regarding the core competencies required for successful healthcare middle managers in Bahrain and Saudi Arabia. The study also sought to explore how these competencies align or differ across hierarchical levels and how the findings can inform the design of competency-based training and development programs for MMs. Understanding these perceptions provides valuable insights into leadership development, capacity building, and workforce planning, and offers a foundation for designing targeted training and competency frameworks tailored to the evolving needs of the region’s healthcare systems. This paper presents the findings of that inquiry and offers recommendations for practice and policy related to healthcare leadership development.

## Methods

### Study Design and Setting

This qualitative descriptive study explored the perceptions of healthcare and medical education managers in the Kingdom of Saudi Arabia (KSA) and the Kingdom of Bahrain (KB) regarding the core competencies required for effective middle managers. The study involved participants from both healthcare institutions and medical education settings to ensure a broad understanding of managerial competencies within these systems.

### Participants and Sampling

A purposive sampling approach was used to select participants who held leadership or managerial positions at three hierarchical levels: senior, middle, and frontline management. This method was chosen to capture diverse and informed perspectives on middle management competencies across organizational tiers.

A total of 27 participants took part in the study which involved (n = 6, 22%), senior managers, (n = 10, 37%), middle managers, and (n = 11, 41%), frontline managers, each representing both the healthcare and medical education sectors (Table 2). Participants included CEOs, deans, heads of clinical or academic departments, and other staff members working under middle managers.

The sample size was determined based on information saturation, which was reached after 25 interviews when no new themes were emerging; two additional interviews confirmed saturation.

Participants were identified through professional networks and institutional contacts who acted as gatekeepers to facilitate access. Invitations were sent via email, providing details of the study purpose, confidentiality, and voluntary participation. No incentives were offered.

The research was approved by Antioch University’s Institutional Review Board and the ethical review boards of those hospitals and medical educational settings in the Kingdoms of Saudi Arabia and Bahrain that participated in the study. Informed consent was obtained from all participants, and strict confidentiality was maintained throughout the study. All data were anonymized, and participants were given the right to withdraw at any stage without any consequences. The study was conducted in accordance with the Declaration of Helsinki.

### Data Collection

Data were collected through semi-structured interviews (Appendix). The questions were adapted from existing literature on healthcare leadership competencies and after structured discussion with two experts. The discussion with experts facilitated the process of item generation and validation including content validity and face validity. Minor linguistic refinements were made following a pilot interview.

Interviews were conducted virtually via Zoom in English and lasted between 15-20 minutes. All interviews were conducted by the author, who maintained a reflexive diary and used neutral probing questions to minimize interviewer bias and hierarchical influence. To ensure accuracy, all sessions were audio-recorded, transcribed verbatim, and cross-checked against the recordings.

### Data Analysis

An inductive thematic analysis was conducted following the six-step framework by Braun and Clarke (2006). This approach was chosen over grounded theory to align with the study’s aim of identifying, rather than generating, theoretical constructs.

The process involved:

1.     Familiarization with transcripts through repeated reading

2.     Initial coding to identify significant statements

3.     Searching for themes by grouping codes

4.     Reviewing themes to ensure coherence

5.     Defining and naming themes based on consensus

6.     Producing the final thematic map

NVivo software was used to organize data and enhance traceability. Credibility was strengthened through peer debriefing, member checking (sharing key themes with five participants for confirmation), and maintaining an audit trail of analytic decisions.

## Results

### Description of Participants

A total of 27 managers were interviewed in this study. Participants’ demographic profiles are detailed in Table 1, which includes data on age, gender, nationality, and years of experience, categorized by management level. A summary analysis of the participants' distribution based on management level, country, and sector is presented in Table 2. Of these participants, 6 were strategic managers (22%), 10 were middle managers (37%), and 11 were frontline managers (41%). Participants represented both the Kingdom of Saudi Arabia (KSA: n = 10, 37%), and the Kingdom of Bahrain (KB: n = 17, 63%), drawn from healthcare (n = 14, 52%) and medical education (n = 13, 48%) institutions.

### Findings from thematic analysis

The dominant themes identified are as follows: 1. Mental ability; 2. Personality; 3. Work ethics; 4. Interaction; 5. Managerial skills; 6. Attitude; 7. Commitment and Responsibility; 8. Ambiguity.

These themes were then used as a framework for the final stage of thematic analysis, known as selective coding. This process helped identify how each theme fit into these categories and allowed for the emergence of five final, overarching themes that encapsulate the key competencies required for successful MMs.

### Personal traits required for successful MMs

Seven major themes were identified as prerequisite personal traits of MMs (Table 3). These were 1. Personality, 2. Agility, 3. Attitude, 4. Managerial Skills, 5. Work Ethics, 6. Mental Ability and 7. Interaction.

The majority of the respondents (n = 18; 67%) identified Personality as the most essential trait for successful middle managers, with integrity and organization as subthemes or components of the trait. For instance, respondent 11 stated that,

“Middle managers are located in a unique position; they have to understand the directives from top management and communicate that to frontline managers, while helping to develop strategies for the effective execution of such institutional goals. It is therefore, important to have the right personality to be able to engage diverse levels of leadership, effective action, and act with integrity while doing so.”

Eleven respondents (41%) identified Managerial Skills as being crucial for successful middle management; subthemes identified under managerial skills included time management and task management.^9^ respondents (33%) identified Work Ethics as an important personal trait for middle managers with leadership quality and teamwork identified as its subthemes. Respondent 2 believed,

“The middle manager must have very high work ethics to be able to cope with the job responsibilities as well as to be able to serve as a role model. So work ethics is linked to leadership ability and the ability to display effective teamwork both working upwards with organizational leaders at the top management level and teamwork working with frontline level managers.”

Eight respondents (30%) identified Mental Ability and Interaction as important personal traits; good communication and active listening were identified as subthemes. 4 respondents (15%) identified Agility and Attitude as desired traits; subthemes identified under Agility included having knowledge and being flexible, while the subtheme identified under Attitude included showing patience.

### Social traits required for successful MMs

The social traits required for successful MMs were identified under five key themes: 1. Personality, 2. Interaction, 3. Work Ethics, 4. Mental Ability, and 5. Commitment.

Social qualities required for middle managers were identified under five themes: Personality, Interaction, Good Work Ethics, Mental Ability, and Commitment. For each of the themes, the subthemes identified varied greatly.  The majority of the respondents (n = 22, 81%) identified the personality of middle managers as an all-important social quality.

**Table 1:** Demographic Profile of Interview Participants

No	Sector	Category	Age	Gender	Nationality	No of yearsof experiencein the field	No of years of experience in the present position
1	Health Care	Frontline Management	42	M	Kingdom of Saudi Arabia	16	16
2	Medical Education	Frontline Management	42	M	Kingdom of Saudi Arabia	13	12
3	Medical Education	Frontline Management	43	M	Kingdom of Saudi Arabia	15	15
4	Medical Education	Frontline Management	32	F	Kingdom of Bahrain	6	1
5	Medical Education	Frontline Management	35	F	Kingdom of Bahrain	3	1
6	Medical Education	Frontline Management	41	M	Kingdom of Bahrain	17	5
7	Health Care	Frontline Management	33	F	Kingdom of Bahrain	7	2
8	Health Care	Frontline Management	44	F	Kingdom of Bahrain	23	1
9	Health Care	Frontline Management	41	M	Kingdom of Bahrain	15	3
10	Health Care	Frontline Management	35	M	Kingdom of Bahrain	8	1
11	Health Care	Frontline Management	35	M	Kingdom of Bahrain	13	4
12	Health Care	Middle Management	41	M	Kingdom of Saudi Arabia	10	10
13	Medical Education	Middle Management	38	M	Kingdom of Saudi Arabia	4	2
14	Medical Education	Middle Management	37	M	Kingdom of Saudi Arabia	8	7
15	Medical Education	Middle Management	37	M	Kingdom of Saudi Arabia	8	7
16	Medical Education	Middle Management	38	F	Kingdom of Bahrain	9	3
17	Health Care	Middle Management	55	F	Kingdom of Bahrain	30	7
18	Health Care	Middle Management	47	F	Kingdom of Bahrain	23	10
19	Health Care	Middle Management	40	F	Kingdom of Bahrain	17	3
20	Health Care	Middle Management	41	M	Kingdom of Bahrain	16	3
21	Medical Education	Middle Management	42	M	Kingdom of Saudi Arabia	8	6
22	Medical Education	Top Management	62	M	Kingdom of Bahrain	35	14
23	Medical Education	Top Management	45	M	Kingdom of Bahrain	14	7
24	Medical Education	Top Management	52	F	Kingdom of Bahrain	27	6
25	Health Care	Top Management	49	F	Kingdom of Bahrain	15	10
26	Health Care	Top Management	42	M	Kingdom of Saudi Arabia	8	6
27	Health Care	Top Management	43	M	Kingdom of Saudi Arabia	17	2

**Table 2:** Distribution of interview participants based on management level and nationality

Participants	Sample size	Medical Education	Healthcare	Kingdom of Bahrain	Kingdom of Saudi Arabia
Senior management	6 (22%)	3 (11%)	3 (11%)	4 (15%)	2 (7%)
Middle management	10 (37%)	5 (19%)	5 (19%)	5 (19%)	5 (19%)
Frontline management	11 (41%)	5 (19%)	6 (22%)	8 (30%)	3 (11%)

**Table 3:** Perceptions regarding personal traits required for MM

Personal traits	Frontline (N =11)n (%)	Middle (N=10)n (%)	Senior (N=6)n (%)	Mean (N=27)n (%)
Personality	10 (90)	9 (90)	6 (100)	25 (93)
Managerial skills	8 (70)	10 (100)	6 (100)	24 (90)
Work ethics	8 (70)	9 (90)	5 (90)	22 (83)
Mental ability	6 (50)	6 (60)	4 (70)	16 (60)
Interaction	7 (60)	6 (60)	3 (50)	16 (60)
Attitude	7 (60)	5 (50)	2 (30)	14 (52)
Agility	3 (30)	6 (60)	3 (50)	12 (45)

Subthemes identified under this quality include humility, being supportive of frontline-level workers, and being honest. As stated by Respondent 10,

“To be a successful middle manager, the person is not only expected to be active, humble, and balanced, but also non-corrupt and supportive. It takes humility to interact successfully with both superiors and junior staffers. The middle manager has to be supportive of both corporate goals as well as supportive of subordinates to help them achieve those goals.”

Eleven respondents (41%) identified Interaction as a key social quality; subthemes identified under this theme were good communication and active listening.^10^ respondents (37%) viewed Good Work Ethics as indispensable for successful middle managers; subthemes identified under this theme included having the ability to motivate, lead, and be a good team player. Other subthemes identified under Interaction were efficiency at team management and teamwork. As observed by Respondent 1,

“A middle manager needs to have good interactional skills. This involves been able to interact well both at the personal level and at the team level. Given that the work of management involves managing a team or group, efficiency at team management and having the know-how to facilitate successful teamwork becomes non-negotiable for the middle manager.”

Five respondents (19%) identified mental ability and commitment as important competencies for successful middle managers. Subthemes identified under mental ability included problem-solving as well as decision making, whereas commitment included a range of sub themes such as being flexible, having patience, and providing health insurance. As stated by Respondent 9,

“The very nature of middle management is such that the role requires high mental ability. It a role where the individual is always solving complex problems and making complex decisions. The ability to engage in problem-solving and decision-making is crucial to success and performance in middle management, particularly under complex circumstances.”

Similarly, another respondent 6 mentioned that,

“Middle managers have to have the mental ability to determine employee needs and factor that into decisions while executing higher organizational programs such as managing health insurance and other benefits programs.”

### Leadership qualities required for successful MMs

Eight themes were identified in respect to the leadership qualities required for successful middle management (Table 5). Eleven respondents (41%) believed in attitude as the essential characteristic of middle managers, 6 respondents (22%) thought personality to be an important competency for middle managers as leaders. Attitude included learning from others, being self-confident, and possessing a desire to grow among others. Personality was defined by being organized and having integrity. Interaction, mental ability, commitment and responsibility, and work ethics were also identified as being critical competences that middle managers must have in order to be effective leaders. These four themes included communication, vision, knowledge, and teamwork, respectively, as one of their sub themes. Interestingly, only 2 respondents (7%) thought managerial skills, which had time and task management as subthemes, as important. As observed by Respondent 7,

“It is imperative that a leader demonstrate high work ethics. This quality not only allows the leader to successfully address their own significant amounts of responsibility, it also serves to influence subordinates and motivate them to perform better.”

Four respondents were not sure of what competency exactly fitted for effective leadership. As one respondent stated,

“Practically speaking, in order to be a successful middle manager, the individual has to be able to execute the assigned roles and responsibilities effectively. Persons who gain the position must have the management skills, knowledge, and educational background to be able to carry out this responsibility. The middle manager has authority over frontline-level managers and employees, so I’m not sure leadership is a separate concept here.”

These domains collectively define the core attributes of successful healthcare middle managers in Bahrain and Saudi Arabia. The findings highlight that effective MMs must balance technical efficiency with interpersonal sensitivity, ethical integrity, and resilience to organizational change.

## Discussion

The findings from the interview reveal seven major personal traits required as prerequisite for MMs: personality, agility, attitude, managerial skills, work ethics, mental ability and interaction. Personality had the highest scores while agility had the lowest. In literature, personality constructs are identified as being critical to both senior and MMs.^19^ The attainment of competency is determined by three factors - personal characteristics, job demands and organizational environment.^20^ Personal characteristics encompass variables such as the individual’s capability, morals, vision and personal philosophy.

**Table 4:** Perceptions regarding social traits required for MM

Social traits	Frontline (N=11)n (%)	Middle (N=10)n (%)	Senior (N=6)n (%)	Mean (N=27)n (%)
Personality	7 (60)	8 (80)	4 (70)	19 (70)
Interaction	7 (60)	8 (80)	4 (60)	19 (70)
Work ethics	4 (40)	7 (70)	4 (70)	15 (55)
Mental Ability	3 (30)	6 (60)	2 (40)	11 (40)
Commitment	2 (20)	6 (60)	2 (40)	10 (37)

**Table 5:** Perceptions regarding leadership qualities required for MM

Leadership qualities	Frontline (N=11)n (%)	Middle (N=10)n (%)	Senior (N=6)n (%)	Mean (N=27)n (%)
Attitude	9 (80)	10 (100)	5 (80)	24 (90)
Personality	8 (70)	9 (90)	5 (90)	22 (83)
Interaction	9 (80)	7 (70)	4 (60)	20 (74)
Mental Ability	4 (40)	7 (70)	4 (70)	15 (55)
Commitment	3 (30)	8 (80)	4 (70)	15 (55)
Responsibility	4 (40)	7 (70)	4 (60)	15 (55)
Work Ethics	4 (40)	7 (70)	4 (60)	15 (55)
Managerial Skills	2 (20)	6 (60)	2 (40)	10 (37)

Job demands includes factors such as career stage, interests, roles, responsibilities and duties. The organizational environment refers to elements such as job culture, climate of the organization, and the economic, political, societal, ecological, and spiritual environment.^20^ The personal characteristics such as capability, morals, vision, personal philosophy as identified in the literature align with, or are closely related to, the personal traits identified in this study: agility, attitude, managerial skills, work ethics, mental ability and interaction.^21^

The sub-themes identified under the personal traits included Integrity, Organization, Time management, Task management, Leadership quality, Teamwork, Decision-making, Vision, Good communication, Active listening, Patience, Knowledge, and Flexibility. These attributes are also supported in the literature.^22^^,^^23^ As observed by Katz, effective leadership involves three types of personal skills - technical, human and conceptual.^13^ Technical skills refer to the knowledge and abilities related to a specific type of work, activity, specialization, or area of competency.^24^ Human skills involve the knowledge and ability to work effectively with people, encompassing competencies such as effective communication, relationship building, fostering team cooperation, creating an atmosphere of trust and psychological safety, and demonstrating sensitivity and concern for the welfare of subordinates.^25^ The managerial competences of time management and task management identified in this study are also supported by literature.^26^^,^^27^ The social qualities required for MMs were identified as humility, good communication, active listening, good work ethics, the ability to motivate, lead and being a good team player. Good communication and relationship-building skills have been discussed as essential personal competences for MMs.^13^ Similarly, social judgment skills- reflecting sound understanding of people and social systems have also been recognized in the literature as critical competencies for successful MMs.^27^

Under leadership qualities required for successful MMs in a medical university or health care organization, our study found a mix of traits and skills which were often used interchangeably by participants. This included self-confidence, a desire to grow, being organized, integrity, communication, vision, knowledge, teamwork, time and task management. As also noted in the literature, effective leadership skills are essential for fostering conditions conducive to teamwork, improving operational outcomes, and ensuring the smooth flow of information.^28^ Specifically, transformational leadership skills have been linked to managerial behavior and managerial effectiveness during transformation.^22^^,^^23^ Additionally, managers who adopt a learning-oriented style have been found to demonstrate high competence in their roles,^29^ particularly within the domain of middle management. Human, technical and conceptual skills have also been identified as important,^13^ although these constructs intercept with already discussed elements such as communication, knowledge, and relationship building.

Problem-solving skills, including the ability to recognize a problem, collect information, and create work plans for problem-solving,^27^ are a core set of managerial skills. Knowledge, defined as the accumulation of information and the possession of a mental framework in which information is organized, is also identified as a vital tool in the middle manager’s portfolio.^27^ The literature further outlines leadership competencies for physicians.^30^ These include team management with the aim of enhancing quality of care through a focus on value, efficiency, and safety,^31^ as well as change management, which is based on the understanding that change is inevitable in the medical sector and that leaders must be equipped to respond effectively to shifting circumstances.^32^^-^^34^

Given that the aim of the study was to identify the differences in the perceptions of frontline, middle and senior managers on the competences required for successful MMs, aggregation of the data based on hierarchical management position is a central to achieving this aim. By analyzing both thematic trends and individual items emerging from the data, the study reveals several insightful patterns. For example, in looking at the findings on personal traits required for MMs, a trend emerged in the data in which senior management tends to emphasize certain traits such as personality, agility, attitude, managerial skills, work ethics, and mental ability more, compared to frontline-level managers. This pattern may reflect senior management’s supervisory role over MMs^35^ granting them a broader perspective. According to them, these qualities enable MMs to implement strategic decisions and fulfill organizational objectives effectively. On the other hand, the frontline management prioritizes interaction and attitude more than both middle and senior management.  This emphasis likely reflects the relational dynamics required for MMs to effectively guide, support, and collaborate with frontline staff.^35^ Confirming this interpretation, sub-themes identified under interaction and attitude were good communication, active listening, and patience, which are attributes that subordinates would appreciate in a senior. These findings suggest that MMs should have a set of personal traits that would allow them to work effectively with both senior and frontline-level management, serving as a vital bridge between strategic leadership and operational execution.

Multiple interpretations are possible when examining the individual attributes identified in the study. For example, both senior and frontline management identified the ability to motivate and ability to lead as one of the most significant characteristics for MMs. While fewer frontline-level managers focused on such attributes, top management highlighted concern and consideration for subordinates as important characteristics. In contrast, frontline management valued interactivity as a key trait for MMs. Representing a mix of all competencies identified by the three levels of management, the management as a whole acknowledges concern for employee well-being, relationship, communication, and being active as crucial competences. Such findings indicate that differences occur in the ways in which respective middle manager skills, competencies or traits may be perceived across the three levels of management, even when all the perceptions and all the items are necessary for successful MMs.

One important competency frequently highlighted in the literature as essential for physician leadership, but notably absent from the study’s findings is cross-cultural sensitivity. In the modern global context, there is strong emphasis on leadership that values diversity, since physicians and nurses tend to interact with patients and families from diverse cultural and social backgrounds.^35^ Accordingly, the current thinking is to instill a strong sense of appreciation for diversity among medical students.^32^ It is likely that this competency was not considered a priority by the research participants in KSA and KB as against competences required for MMs in a global sense.

This study has several strengths. Participants had an average of six years' experience in their current roles, reflecting substantial expertise across senior leadership, middle, and frontline management in healthcare. The sample included 22% from senior management, 37% from middle management, and 41% from frontline management, with the majority representing middle and frontline management. Additionally, participants were nearly evenly split between the medical sector and the broader healthcare sector, supporting the generalizability of findings across both domains. Their roles spanned healthcare delivery, administration, teaching, leadership, research, training, and residency, demonstrating a diverse and rich base of experience informing the study’s insights. The educational background of respondents was also analyzed to contextualize the study's findings.^19^ distinct fields emerged, all within the broader domain of Medical Sciences, including Surgery, Internal Medicine, Family Medicine, Neurology, Neuroscience, Anatomy, Pathology, Radiology, Orthopedic Surgery, Geriatrics, Genetics, Nutrition, Public Health, Epidemiology, and areas such as Hospital Management and Healthcare Governance. The wide disciplinary spread contributes to a rich pool of knowledge and expertise, enhancing the relevance and applicability of the study’s insights.

There are several limitations to this study. All participants were over 31 years old, with the majority in the 41–50 age bracket, and 62% were male. These factors may introduce gender and age-related bias. As with most qualitative research, there is potential for subjective bias during data collection and interpretation. To mitigate this, a structured and well-defined coding process was used, which enhanced consistency and reduced human error. Additionally, the study focused on senior, middle, and frontline-level managers in the Kingdom of Saudi Arabia and Kingdom of Bahrain, limiting the generalizability of findings to other regions.

The findings from this study are useful for understanding perceptions on the leadership and management competencies required for MMs. Since MMs have to work effectively with both senior frontline-level managers in order to successfully deliver organizational goals, an understanding of the perceptions of competencies required for successful MMs across all three levels of management is strategic for successful transformations. The findings may, thus, be applied in the design and delivery of training programs for MMs in health and medical education. The findings from this study also have implications for further research as a portfolio of skills, traits, and competences have been identified for middle management that can be further explored to understand their individual impacts or benefits. Adopting such frameworks can professionalize middle-management roles and enhance continuity between strategic and operational leadership across healthcare organizations globally.

## Conclusion

Middle managers play a pivotal role in translating institutional strategy into effective practice. In Bahrain and Saudi Arabia, success in these roles requires a balanced integration of ethical integrity, technical proficiency, interpersonal competence, and adaptability. Embedding these competencies into leadership-development programs can strengthen healthcare governance, improve communication across managerial tiers, and enhance system responsiveness to reform.

These competencies align with global leadership standards and thus hold relevance for healthcare organizations worldwide. Developing structured, competency-based training frameworks around these domains can enhance leadership capacity, improve organizational performance, and inform future comparative research across regions.

### Conflict of Interest

The authors declare that there is no conflict of interest.

## Supplementary material

Supplementary file 1 Appendix. The Use of Semi-Structured Interviews
